# Global analyses of Chromosome 17 and 18 genes of lung telocytes compared with mesenchymal stem cells, fibroblasts, alveolar type II cells, airway epithelial cells, and lymphocytes

**DOI:** 10.1186/s13062-015-0042-0

**Published:** 2015-03-11

**Authors:** Jian Wang, Ling Ye, Meiling Jin, Xiangdong Wang

**Affiliations:** Department of Pulmonary Medicine, Zhongshan Hospital, Shanghai Institute of Clinical Bioinformatics, Fudan University Center for Clinical Bioinformatics, Biomedical Research Center, Fudan University Medical School, Shanghai, China

**Keywords:** Chromosome 17, Chromosome 18, Genes, Lung, Telocytes, Mesenchymal stem cells, Fibroblasts, Alveolar type II cells, Airwayepithelial cells, Lymphocytes

## Abstract

**Background:**

Telocytes (TCs) is an interstitial cell with extremely long and thin telopodes (Tps) with thin segments (podomers) and dilations (podoms) to interact with neighboring cells. TCs have been found in different organs, while there is still a lack of TCs-specific biomarkers to distinguish TCs from the other cells.

**Results:**

We compared gene expression profiles of murine pulmonary TCs on days 5 (TC5) and days 10 (TC10) with mesenchymal stem cells (MSCs), fibroblasts (Fbs), alveolar type II cells (ATII), airway basal cells (ABCs), proximal airway cells (PACs), CD8^+^ T cells from bronchial lymph nodes (T-BL), and CD8^+^ T cells from lungs (T-LL). The chromosome 17 and 18 genes were extracted for further analysis. The TCs-specific genes and functional networks were identified and analyzed by bioinformatics tools. 16 and 10 of TCs-specific genes were up-regulated and 68 and 22 were down-regulated in chromosome 17 and 18, as compared with other cells respectively. Of them, Mapk14 and Trem2 were up-regulated to indicate the biological function of TCs in immune regulation, and up-regulated MCFD2 and down-regulated E4F1 and PDCD2 had an association with tissue homeostasis for TCs. Over-expressed Dpysl3 may promote TCs self-proliferation and cell-cell network forming.

**Conclusions:**

The differential gene expression in chromosomes 17 and 18 clearly revealed that TCs were the distinctive type of interstitial cells. Our data also indicates that TCs may play a dual role in immune surveillance and immune homoeostasis to keep from immune disorder in acute and chronic pulmonary diseases. TCs also participated in proliferation, differentiation and regeneration.

**Reviewers:**

This article was reviewed by Qing Kay Li and Dragos Cretoiu.

**Electronic supplementary material:**

The online version of this article (doi:10.1186/s13062-015-0042-0) contains supplementary material, which is available to authorized users.

## Background

Telocytes (TCs) have been proposed as a new type of interstitial cells with extremely long telopodes (Tps), which were recognized in different organs, such as trachea and lung [1,2], oesophagus [3], intestine [4], liver [5], kidney [6], heart [7,8], skin [9], eyes [10], urinary tract [11], bladder [12], uterus [13], prostate gland [14], and euromuscular spindles [15], as documented on www.telocytes.com. TCs were found to form a three-dimensional network in close contacts with blood vessels, nerve bundles and cells of the local immune system [8,13,16-18]. There is growing evidence to support that TCs have close associations with stem cells and will be a critical player in regeneration medicine [10,19]. TCs may also take part in immune regulation, proliferation, cellular reparation and fibrosis. However, there are no specific biomarkers to distinguish TCs from other tissue-resident cells. The reliable way to identify TCs is dependent on the specific ultrastructural feature - a small nucleated body and extremely long Tps with podomers and podoms by transmission electron microscopy (TEM) [20]. TCs present positive CD34, c-kit and vimentin by immunohistochemistry [19] and other markers like iNOS, caveolin-1, VEGF-D and PDGFRα were also identified to indicate various immunophetypes of TCs existed in different organs [4,21].

Our previous study initially approved the existence of TCs in the trachea and lung tissues of the mouse and human [1,22,23]. We compared genes expression profiles of murine TCs with mesenchymal stem cells (MSCs) and fibroblasts (Fbs) and proteomic profiles of human lung TCs and Fbs to explore TC-specific markers and their biological function [24,25]. MicroRNA signature was also used to differentiate TCs from other interstitial cells [26]. Furthermore, we revealed features and patterns of genes in chromosomes 1 by comparing gene expression profiles of murine pulmonary TCs, MSCs, Fbs, alveolar type II cells (ATII), airway basal cells (ABCs), proximal airway cells (PACs), CD8^+^ T cells from bronchial lymph nodes (T-BL), and CD8^+^ T cells from lungs (T-LL) by global analyses [27]. The similar work has been done to detect patterns of TCs-specific genes in chromosome 2 and 3, and showed that TCs played an important role in tissue injury and aging, inhibition of tissue inflammation, tumor promotion, and development of pulmonary fibrosis and other interstitial lung diseases [28]. However, features and patterns of TCs-specific genes in other chromosomes have not been uncovered, and potential function of TCs still remained unclear.

The present study aimed at investigating features and patterns of TCs-specific gene profiles and exploring potential function of TCs by focusing in chromosomes 17 and 18. The significant difference in gene expression profiles of murine pulmonary TCs on days 5 (TC5) and days 10 (TC10) with the other cells, MSCs, Fbs, ATII, ABCs, PACs, T-BL and T-LL were compared to identify TCs-specific genes and the functional networks were identified by bioinformatics tools.

## Methods

### Cell sampling and data collection

Gene expression profiles of murine pulmonary TCs on days 5 and 10, MSCs and Fbs were obtained from our previous study [24]. TCs were isolated from BALB/c mouse lung tissue and cultured for 5 and 10 days, respectively. MSCs and Fbs were obtained from Sciencell Research Laboratories (M7500-57, Carlsbad, CA, USA) and Chinese Academy of Science (GNM28, Shanghai, China), respectively. ATII, ABCs, PACs, T-BL, T-LL gene expression profiles were obtained from the National Center for Biotechnology Information (NCBI) Gene Expression Omnibus database (GSE6846, GSE27379, GSE28651) [29]. Alveolar type II cells (GSM157835-GSM157837) were isolated from 8 weeks old sex age matched littermate control mice. CD8^+^ T-cells from lungs and bronchial lymph nodes (GSM677065) and CD8^+^ T-cells from bronchial lymph nodes, lymphocytes (GSM677065) derived from mice (CL4). Murine proximal airway duct (GSM709834-GSM709836) and basal cells (GSM709832, GSM709833) were isolated from 8–12 week old C57BL/6 mice.

### Data mining and preprocessing

The gene expression profiles of murine lung TCs, MSCs and Fbs from our previous study contained 23861 probes. About 13236 probes and 11532 genes were defined after eliminating the probes without corresponding official symbols. Gene expression of ATII, ABCs, PACs, T-BL, and T-LL from GEO originally included 45,101 probes. 5684 probes without corresponding official symbols were eliminated and 39,417 probes and 21,680 genes were obtained. Data integration and genes detected in all the samples were selected to analysis, of which 11532 genes were elected finally. Totally, 582 genes of the chromosome 17 and 267 genes of the chromosome 18 were focused and furthermore analyzed in the present study.

### Identification of differentially expressed genes

Gene expression data were normalized and imported into Agilent GeneSpring GX software (version 11.5.1) for further analysis. Differentially expressed genes between two samples were identified through Fold Change filtering as our previous research [27]. The propensity of functional changes was reflected in different levels of the gene expression in each cell types. The genes in TCs on days 5 and 10, which were up- or down-regulated more than one-fold compared with other cells, were identified as TCs-specific genes in the present study. Up- or down-regulated folds of TCs genes were calculated by comparing with other cells, after the averages of gene expression in cells were obtained from the raw data of multi-databases, as shown in Additional files 1 and 2.

## Results

Fifty three genes were up-regulated and 92 down-regulated in chromosome 17 of TC5 as compared with other cells, and 28 genes were up-regulated and 167 down-regulated in TC10. Of genes up or down-regulated at TC5 and TC10, 16 over-expressed genes (i.e. 2900073G15Ri, Ccnd3, Chtf18, Clic1, Fem1a, Fez2, Kifc1, Mapk14, Mcfd2, Mtch1, Pgp, Tbcc, Tubb4, Tubb5, Zfand3, Trem2) were obtained in chromosome 17 of TCs. Among them, Trem2 was up-regulated over five folds in TC5 and TC10, as compared with others. Sixty eight genes were down-regulated in TC5 and TC10, of which C030034I22Rik was expressed in TCs 2–5 folds lower than in other cells (Tables 1, 2, 3 and 4). In chromosome 18, we found 22 up-regulated genes and 31 down-regulated genes in TC5 and 15 up-regulated genes and 57 down-regulated genes in TC10 as compared with other cells. Eleven genes (9430020K01Rik, Bin1, Cdh2, Fech, Txnl4a, Usp14, Yipf5, Dpysl3, Lims2, Tubb6) were up-regulated and 22 genes down-regulated (fold >1). Among them, Dpysl3, Lims2 and Tubb6 were up-regulated 2–5 folds in TC5 and TC10, while Scgb3a2 and Zfp397 was expressed in TCs 2–5 folds lower than other cells (Tables 5, 6, 7 and 8).

**Table 1:** **Summary of genes expressed preferentially in TCs, as compared with others in chromosome 17**

**Compaired pairs/fold up-regulated**	**>1**	**>2**	**>5**
TC5 vs. others	47	4	2
TC10 vs. others	22	4	2
TCs vs. others	15	0	1

**Table 2:** **Summary of genes expressed preferentially in TCs, as compared with others in chromosome 17**

**Compaired pairs/fold down-regulated**	**>1**	**>2**	**>5**
TC5 vs. others	83	9	0
TC10 vs. others	146	21	0
TCs vs. others	67	1	0

**Table 3:** **The up-regulated fold of specific genes in TCs as compared with others in chromosome 17**

**Folds(TC5 vs. others/TC10 vs. others)**							
**(A) Genes up-regulated between one- and twofold in TCs as compared with others**							
**Gene symbol**	**Fibroblast**	**Stem**	**ATII**	**CD8_T_BL**	**CD8_T_LL**	**Basal_cell**	**Duct_cell**
2900073G15Rik	2.90/1.87	1.81/1.17	2.81/1.81	4.89/3.16	3.79/2.45	3.70/2.45	3.45/2.22
Ccnd3	2.49/2.33	2.37/2.22	1.40/1.31	1.47/1.38	1.73/1.62	2.44/1.62	5.29/4.96
Chtf18	2.41/1.86	1.45/1.12	4.34/3.35	2.24/1.72	12.43/9.59	5.69/9.59	2.39/1.84
Clic1	3.64/2.26	2.10/1.30	2.01/1.25	2.37/1.47	3.10/1.93	2.45/1.93	1.94/1.21
Fem1a	1.99/1.67	1.86/1.56	2.24/1.87	6.71/5.62	7.09/5.94	1.22/5.94	1.31/1.10
Fez2	3.14/2.86	1.87/1.71	9.51/8.69	3.84/3.51	4.21/3.85	3.11/3.85	3.49/3.19
Kifc1	2.23/2.42	1.10/1.19	8.53/9.27	1.91/2.08	1.05/1.14	5.49/1.14	4.52/4.91
Mapk14	1.57/1.02	1.71/1.11	7.98/5.19	3.38/2.20	3.96/2.57	1.96/2.57	2.63/1.71
Mcfd2	1.04/1.03	1.38/1.37	1.24/1.23	2.88/2.85	3.35/3.31	6.02/3.31	4.26/4.22
Mtch1	2.24/1.63	1.66/1.20	2.73/1.98	88.12/64.07	144.31/104.92	35.78/104.92	27.26/19.82
Pgp	1.25/1.20	1.72/1.65	1.50/1.44	2.85/2.73	2.99/2.86	3.01/2.86	4.71/4.51
Tbcc	2.63/2.04	1.97/1.53	2.34/1.82	2.85/2.21	2.84/2.20	1.81/2.20	1.87/1.45
Tubb4	4.41/3.16	1.77/1.27	4.23/3.04	13.43/9.63	40.35/28.94	8.72/28.94	2.66/1.90
Tubb5	3.95/2.55	1.76/1.13	5.87/3.79	2.81/1.82	4.11/2.66	4.28/2.66	3.33/2.15
Zfand3	1.53/1.26	1.47/1.21	4.12/3.39	3.50/2.89	5.50/4.53	4.37/4.53	5.48/4.51
**(B) Genes up-regulated between five- and tenfold in TCs as compared with others**							
Trem2	15.11/76.80	36.31/184.56	30.83/156.68	25.98/132.03	51.13/259.88	30.45/259.88	44.38/225.54

**Table 4:** The down-regulated fold of specific genes in TCs as compared with others in chromosome 17

**Folds(TC5 vs others/TC10 vs others)**							
**(A) Genes up-regulated between one- and twofold in TCs as compared with others**							
**Gene symbol**	**Fibroblast**	**Stem**	**ATII**	**CD8_T_BL**	**CD8_T_LL**	**Basal_cell**	**Duct_cell**
1600014C23Rik	2.69/3.97	1.21/1.78	12.47/18.43	2.66/3.93	2.55/3.77	9.29/13.72	7.04/10.40
2310061I04Rik	1.54/1.80	1.27/1.49	6.68/7.82	3.66/4.29	3.06/3.58	6.36/7.44	6.74/7.89
4930539E08Rik	1.40/1.69	1.07/1.29	5.65/6.83	31.25/37.81	38.16/46.16	32.79/39.68	24.54/29.69
5430411C19Rik	2.30/2.15	1.13/1.05	3.40/3.18	23.14/21.65	15.46/14.47	7.61/7.12	14.76/13.81
A630033E08Rik	2.67/3.15	1.24/1.46	7.91/9.35	29.81/35.22	41.89/49.49	20.02/23.65	6.67/7.88
Atl2	1.32/1.84	2.22/3.10	21.29/29.73	14.23/19.88	13.07/18.25	33.45/46.71	32.74/45.72
Atp6v1g2	1.05/1.44	1.72/2.36	2.20/3.02	1.80/2.47	1.61/2.20	2.38/3.26	3.17/4.34
BC003965	2.62/2.97	1.70/1.93	8.77/9.92	21.82/24.69	17.18/19.44	39.62/44.82	25.30/28.62
BC011426	1.06/1.32	1.25/1.56	5.65/7.04	15.51/19.30	18.28/22.76	1.50/1.86	3.09/3.85
BC051142	1.12/1.60	1.16/1.67	1.38/1.98	2.89/4.15	1.45/2.08	3.18/4.58	2.00/2.87
Cdc5l	1.79/1.69	2.19/2.08	171.49/162.64	269.71/255.80	210.90/200.03	271.87/257.85	238.68/226.37
Clpp	1.05/1.06	1.63/1.64	6.30/6.34	4.95/4.97	3.45/3.47	8.03/8.08	7.75/7.79
Cox7a2l	1.42/2.18	1.21/1.87	1.41/2.17	3.45/5.30	2.34/3.60	4.80/7.39	3.91/6.02
Dpp9	1.33/1.63	1.44/1.76	18.97/23.22	8.31/10.17	5.90/7.22	3.96/4.84	2.59/3.17
Dpy30	1.38/2.23	1.11/1.78	6.93/11.15	12.89/20.76	7.90/12.73	16.78/27.02	15.21/24.49
E4f1	1.60/1.63	2.12/2.16	2.86/2.92	14.41/14.68	23.06/23.50	3.05/3.11	2.74/2.79
Eml4	1.08/1.61	4.20/6.27	1.20/1.79	2.43/3.63	2.77/4.15	2.32/3.47	2.45/3.66
Gabbr1	1.09/1.34	3.45/4.23	9.29/11.39	29.00/35.58	35.59/43.67	19.70/24.17	12.19/14.96
Glo1	3.48/4.60	1.46/1.93	3.77/4.98	3.69/4.87	2.29/3.03	4.37/5.77	6.12/8.08
Gm88	1.52/1.02	2.10/1.41	52.54/35.36	11.10/7.47	8.02/5.40	73.00/49.13	27.98/18.83
H2-M3	1.86/1.96	3.10/3.27	4.80/5.07	50.87/53.67	44.63/47.09	10.46/11.04	6.11/6.45
Hn1l	2.75/4.64	2.51/4.24	6.48/10.95	2.83/4.79	1.18/2.00	4.37/7.39	7.69/13.00
Hsf2bp	4.35/4.89	1.34/1.50	4.61/5.18	4.61/5.19	7.26/8.16	7.73/8.69	14.52/16.33
Khsrp	1.29/1.72	1.16/1.55	2.24/2.99	3.88/5.18	3.32/4.43	16.48/22.01	13.07/17.45
Mdc1	1.52/1.72	2.00/2.26	3.04/3.45	2.69/3.05	2.71/3.07	4.07/4.60	2.42/2.74
Mettl4	1.26/1.67	1.32/1.75	1.34/1.77	3.57/4.74	3.75/4.98	7.81/10.37	4.95/6.57
Mrps18a	2.16/3.03	1.25/1.75	2.71/3.80	1.90/2.67	1.52/2.14	3.50/4.91	3.08/4.32
Msh6	1.26/1.82	2.07/3.00	1.92/2.78	3.44/4.99	2.42/3.51	3.41/4.94	2.44/3.53
Ndufv2	1.76/2.27	1.52/1.96	1.42/1.82	1.64/2.11	1.59/2.05	2.38/3.06	2.02/2.60
Pdcd2	1.57/1.98	2.66/3.36	1.83/2.31	3.34/4.22	2.81/3.55	2.83/3.58	2.09/2.65
Pdpk1	1.51/2.36	1.27/1.99	1.17/1.83	3.12/4.88	2.17/3.41	1.46/2.29	1.74/2.72
Phf1	1.10/1.17	1.92/2.04	19.66/20.88	6.46/6.86	9.04/9.61	4.90/5.21	3.46/3.67
Pknox1	1.27/1.17	1.89/1.75	21.31/19.71	25.53/23.61	19.15/17.71	25.79/23.86	24.24/22.42
Polr1c	1.49/2.18	1.37/2.00	9.61/14.04	7.75/11.33	6.96/10.18	11.71/17.11	9.91/14.48
Prrt1	2.80/2.43	1.92/1.67	6.39/5.55	6.29/5.46	11.82/10.27	2.39/2.08	1.41/1.23
Psors1c2	6.73/5.81	1.31/1.13	12.72/10.98	13.83/11.94	12.50/10.79	8.46/7.30	8.08/6.98
Rab5a	1.08/1.42	1.34/1.75	4.47/5.86	7.74/10.15	8.45/11.08	3.48/4.56	3.64/4.78
Rdbp	1.22/1.55	1.48/1.88	1.93/2.45	2.51/3.18	1.42/1.80	4.19/5.32	3.61/4.58
Ring1	1.28/1.35	1.05/1.11	59.34/62.76	20.66/21.85	3.77/3.99	92.26/97.58	73.81/78.07
Rnps1	1.49/1.83	1.67/2.05	20.20/24.85	60.50/74.42	53.28/65.54	44.57/54.83	47.99/59.03
Skiv2l	1.44/1.33	1.55/1.44	25.93/24.00	24.77/22.93	20.72/19.18	4.41/4.09	3.28/3.04
Slc22a1	1.79/2.00	1.58/1.76	7.12/7.94	1.22/1.36	1.48/1.65	11.28/12.57	11.06/12.33
Slc25a23	1.03/1.45	1.28/1.79	44.78/62.78	3.84/5.38	2.57/3.61	40.36/56.58	32.46/45.51
Solh	1.21/1.22	1.24/1.24	88.56/89.01	148.74/149.50	153.65/154.43	111.96/112.53	76.12/76.51
Srd5a2	1.05/1.37	1.09/1.43	2.87/3.76	6.38/8.38	2.32/3.05	5.92/7.78	4.08/5.36
Srrm2	1.13/1.54	1.79/2.42	24.93/33.79	39.53/53.59	62.88/85.24	31.91/43.25	19.68/26.67
Stk38	1.55/1.43	1.09/1.00	10.55/9.75	64.22/59.33	56.48/52.17	28.77/26.58	12.08/11.16
Supt3h	1.91/1.52	2.66/2.12	15.28/12.16	16.32/12.98	12.85/10.22	49.56/39.43	27.98/22.27
Sytl3	1.15/1.28	1.06/1.19	4.59/5.14	4.63/5.18	6.65/7.44	10.58/11.85	5.56/6.23
Taf8	1.47/1.50	1.87/1.90	2.86/2.90	3.23/3.28	2.51/2.55	4.39/4.45	2.28/2.31
Tap1	3.12/2.15	1.91/1.32	25.60/17.68	173.22/119.61	180.68/124.76	46.31/31.97	36.19/24.99
Tbc1d5	1.03/1.16	1.53/1.72	1.16/1.31	1.18/1.33	1.32/1.49	2.09/2.35	1.13/1.27
Tbp	1.01/1.45	2.31/3.33	1.11/1.60	2.79/4.02	1.77/2.56	1.87/2.70	1.09/1.58
Tcf19	1.65/2.21	1.26/1.68	2.05/2.74	3.70/4.95	2.53/3.38	2.89/3.86	2.89/3.87
Telo2	1.35/1.69	1.69/2.13	1.35/1.70	1.43/1.80	1.35/1.69	4.90/6.16	3.84/4.83
Thumpd2	1.66/1.62	1.38/1.35	1.52/1.48	4.88/4.77	4.47/4.37	5.39/5.27	2.68/2.62
Wdr27	1.30/1.15	1.28/1.13	8.01/7.09	2.61/2.31	3.82/3.38	15.14/13.40	1.80/1.60
Wdr43	1.08/1.44	3.01/4.00	1.89/2.52	3.40/4.52	3.56/4.73	4.38/5.83	6.09/8.10
Zfp101	3.45/4.24	1.27/1.56	3.57/4.39	14.70/18.07	15.77/19.38	6.56/8.06	2.51/3.09
Zfp160	1.94/2.10	1.67/1.81	2.14/2.32	14.45/15.68	10.13/10.99	2.96/3.22	2.67/2.90
Zfp161	1.70/1.76	1.71/1.77	5.14/5.32	10.17/10.51	10.33/10.67	14.79/15.28	4.90/5.07
Zfp318	1.11/1.54	2.00/2.77	1.53/2.11	3.15/4.36	2.63/3.65	4.30/5.95	2.39/3.31
Zfp472	1.85/2.12	1.17/1.34	4.32/4.94	45.25/51.74	33.07/37.81	2.46/2.82	1.59/1.82
Zfp54	1.24/1.86	1.08/1.61	1.75/2.63	5.39/8.08	7.68/11.52	1.90/2.85	1.47/2.20
Zfp563	2.16/2.00	1.39/1.29	5.71/5.28	9.05/8.37	4.10/3.79	12.25/11.33	6.19/5.72
Zfp677	1.32/2.15	1.02/1.66	2.43/3.96	12.31/20.09	14.11/23.02	2.32/3.79	1.10/1.80
Znrd1	1.29/1.64	1.17/1.49	1.82/2.31	2.95/3.74	2.57/3.26	1.88/2.39	1.61/2.04
**(B) Genes down-regulated between two- and fivefold in TCs as compared with others**							
C030034I22Rik	5.83/6.02	3.09/3.18	3.61/3.72	26.28/27.11	28.59/29.49	10.78/11.11	5.35/5.52

**Table 5:** **Summary of genes expressed preferentially in TCs, as compared with others in chromosome 18**

**Compaired pairs/fold up-regulated**	**>1**	**>2**	**>5**
TC5 vs. others	14	8	0
TC10 vs. others	12	3	0
TCs vs. others	7	3	0

**Table 6:** **Summary of genes expressed preferentially in TCs, as compared with others in chromosome 18**

**Compaired pairs/fold down-regulated**	**>1**	**>2**	**>5**
TC5 vs. others	28	3	0
TC10 vs. others	50	6	1
TCs vs. others	20	2	0

**Table 7:** **The up-regulated fold of specific genes in TCs as compared with others in chromosome 18**

**Folds(TC5 vs others/TC10 vs others)**							
**(A) Genes up-regulated between one- and twofold in TCs as compared with others**							
**Gene symbol**	**Fibroblast**	**Stem**	**ATII**	**CD8_T_BL**	**CD8_T_LL**	**Basal_cell**	**Duct_cell**
9430020K01Rik	1.16/1.02	1.85/1.64	1.47/1.30	24.11/21.33	55.70/49.26	5.37/4.75	6.55/5.79
Bin1	4.38/3.55	1.57/1.28	10.40/8.44	13.96/11.33	11.95/9.70	20.18/16.37	20.95/16.99
Cdh2	167.95/180.20	1.55/1.67	86.02/92.30	144.75/155.30	24.65/26.45	6.56/7.04	13.85/14.86
Fech	1.44/1.39	1.93/1.87	1.53/1.48	2.63/2.54	3.01/2.91	2.52/2.43	2.80/2.71
Txnl4a	2.14/1.46	1.78/1.21	2.04/1.39	2.85/1.94	2.95/2.01	2.74/1.87	2.84/1.94
Usp14	2.14/1.40	1.97/1.29	2.61/1.72	2.01/1.32	2.37/1.55	3.88/2.55	3.33/2.19
Yipf5	1.17/1.04	2.22/1.96	1.78/1.57	1.98/1.75	3.09/2.73	2.12/1.87	2.77/2.44
**(B) Genes down-regulated between two- and fivefold in TCs as compared with others**							
Dpysl3	226.34/161.65	4.86/3.47	35.54/25.38	220.54/157.51	38.85/27.75	2.99/2.13	17.08/12.20
Lims2	2.16/2.42	28.92/32.46	6.04/6.77	119.74/134.38	174.63/195.98	37.64/42.24	393.49/441.60
Tubb6	5.38/3.70	3.96/2.73	25.62/17.62	457.27/314.51	267.70/184.12	16.55/11.38	9.71/6.68

**Table 8:** **The down-regulated fold of specific genes in TCs as compared with others in chromosome 18**

**Folds(TC5 vs others/TC10 vs others)**							
**(A) Genes up-regulated between one- and twofold in TCs as compared with others**							
**Gene symbol**	**Fibroblast**	**Stem**	**ATII**	**CD8_T_BL**	**CD8_T_LL**	**Basal_cell**	**Duct_cell**
1700034H14Rik	2.01/2.59	1.13/1.46	2.11/2.72	11.70/15.08	10.71/13.80	6.98/9.00	5.12/6.59
2210409D07Rik	1.00/1.54	1.01/1.55	4.12/6.31	3.73/5.72	6.93/10.63	16.66/25.54	8.70/13.34
5430411K18Rik	1.94/2.09	1.26/1.36	2.71/2.92	4.24/4.56	4.50/4.85	2.92/3.15	2.54/2.74
Arhgap12	1.08/1.58	3.11/4.55	1.29/1.89	2.58/3.78	3.17/4.64	10.67/15.62	6.19/9.06
Arhgap26	1.13/1.17	1.49/1.54	8.78/9.06	18.96/19.57	13.15/13.57	70.65/72.91	35.92/37.07
Arsi	1.07/1.18	1.14/1.26	25.60/28.26	21.69/23.95	56.02/61.85	43.99/48.58	17.26/19.06
Hspa9	2.83/3.57	2.15/2.70	1.08/1.36	1.38/1.74	1.43/1.80	1.82/2.30	2.93/3.69
Impa2	3.99/4.10	1.36/1.39	1.30/1.34	1.65/1.69	1.66/1.71	2.94/3.02	3.17/3.26
Iws1	2.27/2.98	1.52/2.00	3.88/5.11	12.77/16.79	13.43/17.66	10.93/14.36	7.67/10.08
Map3k2	1.48/1.47	2.36/2.34	3.77/3.74	9.79/9.72	11.64/11.55	9.24/9.17	6.01/5.96
Mapk4	1.08/1.16	1.06/1.13	4.58/4.89	4.69/5.01	6.72/7.17	3.73/3.98	8.17/8.71
Matr3	2.04/3.30	1.17/1.89	8.32/13.47	19.84/32.15	23.19/37.58	7.76/12.57	9.33/15.11
Nfatc1	2.44/1.73	1.56/1.11	16.01/11.35	35.31/25.03	34.71/24.61	4.90/3.47	4.92/3.49
Pcdhb10	1.92/2.19	1.55/1.77	5.62/6.41	1.55/1.77	15.45/17.62	1.47/1.67	1.95/2.23
Pcdhb15	2.23/1.77	3.33/2.65	5.09/4.05	3.80/3.02	6.70/5.34	1.60/1.28	4.69/3.74
Pcdhb8	1.96/2.06	1.27/1.33	2.23/2.34	12.19/12.82	5.11/5.38	6.13/6.45	6.10/6.42
Seh1l	1.63/2.35	1.53/2.21	1.74/2.51	1.19/1.71	1.50/2.16	5.80/8.39	5.60/8.10
Snx24	2.08/1.96	1.10/1.04	131.64/124.43	1.36/1.29	1.71/1.62	84.17/79.56	26.83/25.36
Stard6	2.97/1.83	1.71/1.05	10.62/6.54	11.65/7.18	14.98/9.23	7.01/4.32	18.24/11.24
Tmed7	1.92/1.86	1.34/1.30	31.81/30.96	20.57/20.03	22.11/21.53	31.42/30.59	25.99/25.30
**(B) Genes down-regulated between two- and fivefold in TCs as compared with others**							
Scgb3a2	2.14/3.14	2.16/3.17	4083.95/5996.46	2.25/3.30	6.29/9.24	6608.77/9703.65	3851.22/5654.77
Zfp397	2.34/3.46	2.52/3.71	2.24/3.31	2.78/4.11	2.89/4.26	8.71/12.84	3.70/5.46

In chromosome 17 the number of up- and down-regulated genes more than one-fold in TC5 was 332 and 250, 330 and 252, 184 and 398, 232 and 350, 223 and 359,153 and 429, or 192 and 390, as compared with MSCs, Fbs, ATII, ABCs, PACs, T-BL or T-LL, respectively. The number of up- and down-regulated genes more than one-fold in TC10 was 226 and 356, 245 and 337, 139 and 443, 194 and 388,177 and 405, 120 and 462, or 146 and 436, as compared with MSCs, Fbs, ATII, ABCs, PACs, T-BL or T-LL, respectively. The number of up- and down-regulated genes in TC5 and TC10 was 220 and 244, 229 and 236, 134 and 393, 185 and 341,170 and 352, 112 and 421, or 142 and 386, as compared with MSCs, Fbs, ATII, ABCs, PACs, T-BL or T-LL, respectively (Table 9). In chromosome 18 the number of up- and down-regulated genes in TC5 was 190 and 77, 173 and 94, 73 and 194, 92 and 175, 85 and 182, 54 and 213, or 58 and 209, as compared with MSCs, Fbs, ATII, ABCs, PACs, T-BL or T-LL, respectively. The number of up- and down-regulated genes in TC10 was 152 and 115, 140 and 127, 58 and 209, 69 and 198, 74 and 193, 44 and 223, or 49 and 218, as compared with MSCs, Fbs, ATII, ABCs, PACs, T-BL or T-LL, respectively. The number of up- and down-regulated genes in TC5 and TC10 was 143 and 68, 130 and 84, 52 and 188, 66 and 172, 69 and 177, 41 and 210, or 48 and 208, as compared with MSCs, Fbs, ATII, ABCs, PACs, T-BL or T-LL, respectively (Table 10). The detailed genes were shown in Additional files 3 and 4.

**Table 9:** **The number of genes specifically up- or down-regulated in pulmonary telocytes, as compared with other cells respectively in chromosome 17**

**Compaired pairs**	**Up > 1**	**Up > 2**	**Up > 5**	**Down > 1**	**Down > 2**	**Down > 5**	**Down > 10**
TC5 vs. stem	332	106	34	250	75	10	3
TC10 vs. stem	226	90	25	356	132	25	6
TCs vs. stem	220	68	19	244	68	8	3
TC5 vs. fibroblast	330	136	50	252	87	14	3
TC10 vs. fibroblast	245	95	36	337	124	22	4
TCs vs. fibroblast	229	84	31	236	79	9	2
TC5 vs. ATII	184	97	28	398	283	160	92
TC10 vs. ATII	139	71	19	443	333	193	114
TCs vs. ATII	134	61	15	393	227	151	85
TC5 vs. CD8BL	232	139	60	350	269	160	105
TC10 vs. CD8BL	194	113	40	388	300	175	115
TCs vs. CD8BL	185	100	37	341	261	149	97
TC5 vs. CD8LL	223	135	55	359	262	158	104
TC10 vs. CD8LL	177	105	43	405	301	170	122
TCs vs. CD8LL	170	99	39	352	254	150	95
TC5 vs. basal cell	153	77	28	429	332	201	120
TC10 vs. basal cell	120	59	23	462	377	238	150
TCs vs. basal cell	112	55	16	421	327	194	111
TC5 vs. duct cell	192	100	36	390	288	169	98
TC10 vs. duct cell	146	75	25	436	332	205	119
TCs vs. duct cell	142	67	20	386	282	165	88

**Table 10:** **The number of genes specifically up- or down-regulated in pulmonary telocytes, as compared with other cells respectively in chromosome 18**

**Compaired pairs**	**Up > 1**	**Up > 2**	**Up > 5**	**Down > 1**	**Down > 2**	**Down > 5**	**Down > 10**
TC5 vs. stem	190	83	18	77	21	0	0
TC10 vs. stem	152	49	15	115	31	4	1
TCs vs. stem	143	42	9	68	17	0	0
TC5 vs. fibroblast	173	81	17	94	31	1	1
TC10 vs. fibroblast	140	46	11	127	45	5	1
TCs vs. fibroblast	130	44	10	84	24	0	0
TC5 vs. ATII	73	35	14	194	148	77	43
TC10 vs. ATII	58	25	12	209	165	99	56
TCs vs. ATII	52	22	11	188	146	73	41
TC5 vs. CD8BL	92	50	27	175	133	84	49
TC10 vs. CD8BL	69	42	20	198	153	94	59
TCs vs. CD8BL	66	36	18	172	131	80	47
TC5 vs. CD8LL	85	59	23	182	142	94	46
TC10 vs. CD8LL	74	39	18	193	160	104	63
TCs vs. CD8LL	69	37	17	177	138	90	45
TC5 vs. basal cell	54	31	10	213	178	116	66
TC10 vs. basal cell	44	23	9	223	194	140	84
TCs vs. basal cell	41	19	8	210	175	112	63
TC5 vs. duct cell	58	37	11	209	167	106	54
TC10 vs. duct cell	49	27	11	218	186	126	70
TCs vs. duct cell	48	22	9	208	164	102	52

**Figure 1:**
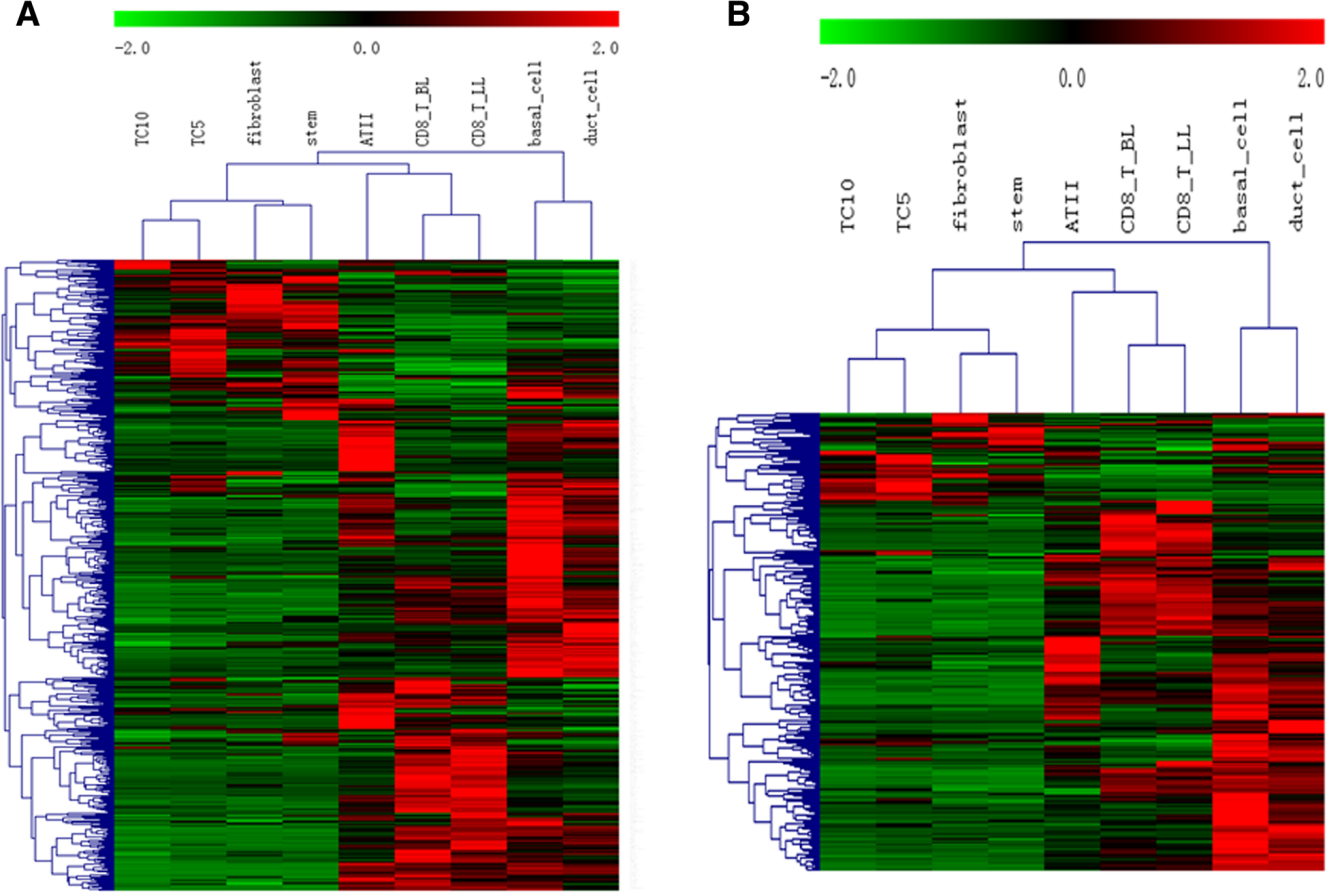
**Hierarchical clustering of 582 genes in chromosomes 17 (A) and 267 genes 18 (B) among murine pulmonary telocytes (TCs), mesenchymal stem cells (MSCs), fibroblasts (Fbs), alveolar type II cells (ATII), airwaybasal cells (ABCs), proximal airway cells (PACs), CD8** ^**+**^ **T cells from bronchial lymph nodes (T-BL) and CD8** ^**+**^ **T cells from lungs (T-LL).**

**Figure 2:**
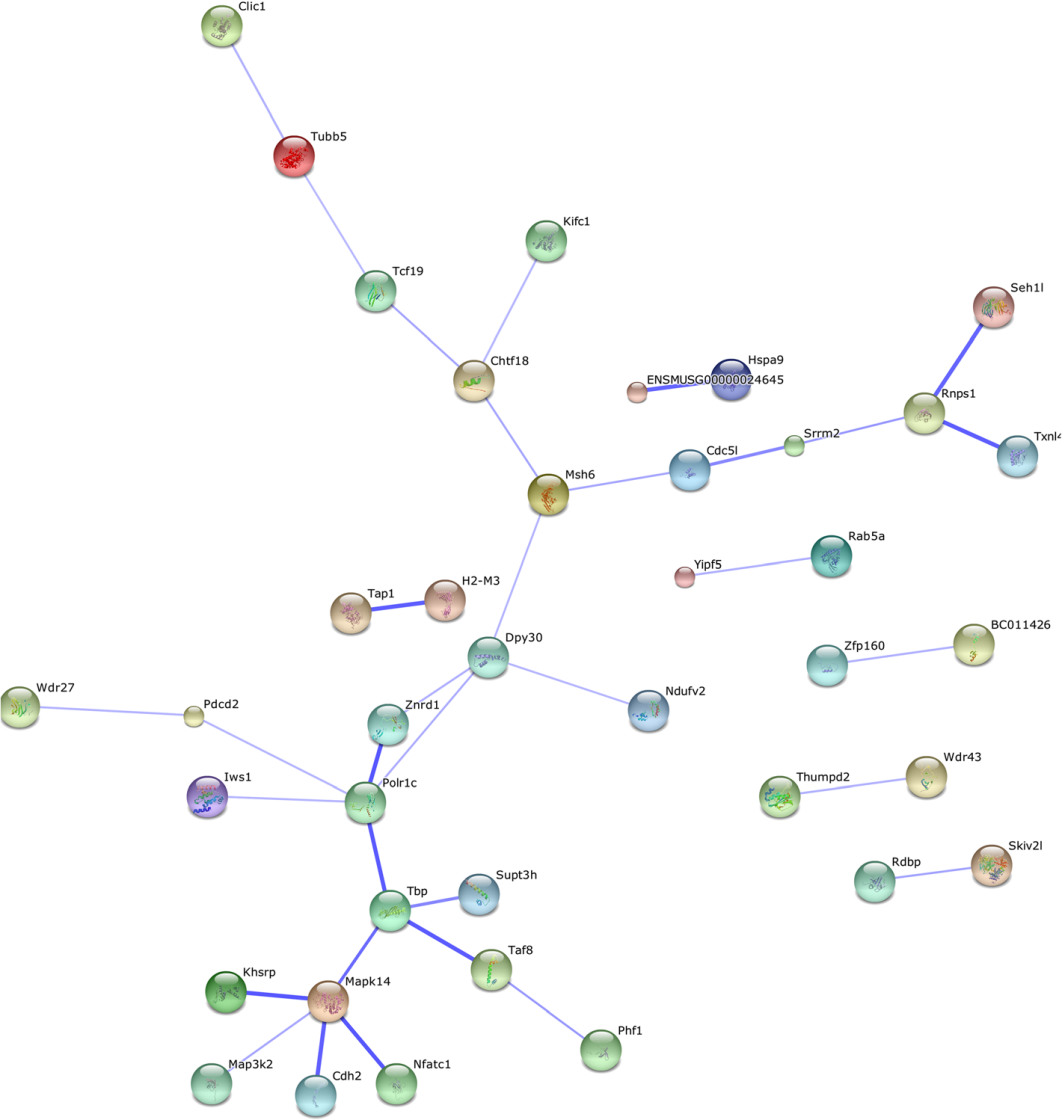
**The physical and functional interaction of specific genes by String Network analysis in chromosome 17 and 18.**

**Figure 3:**
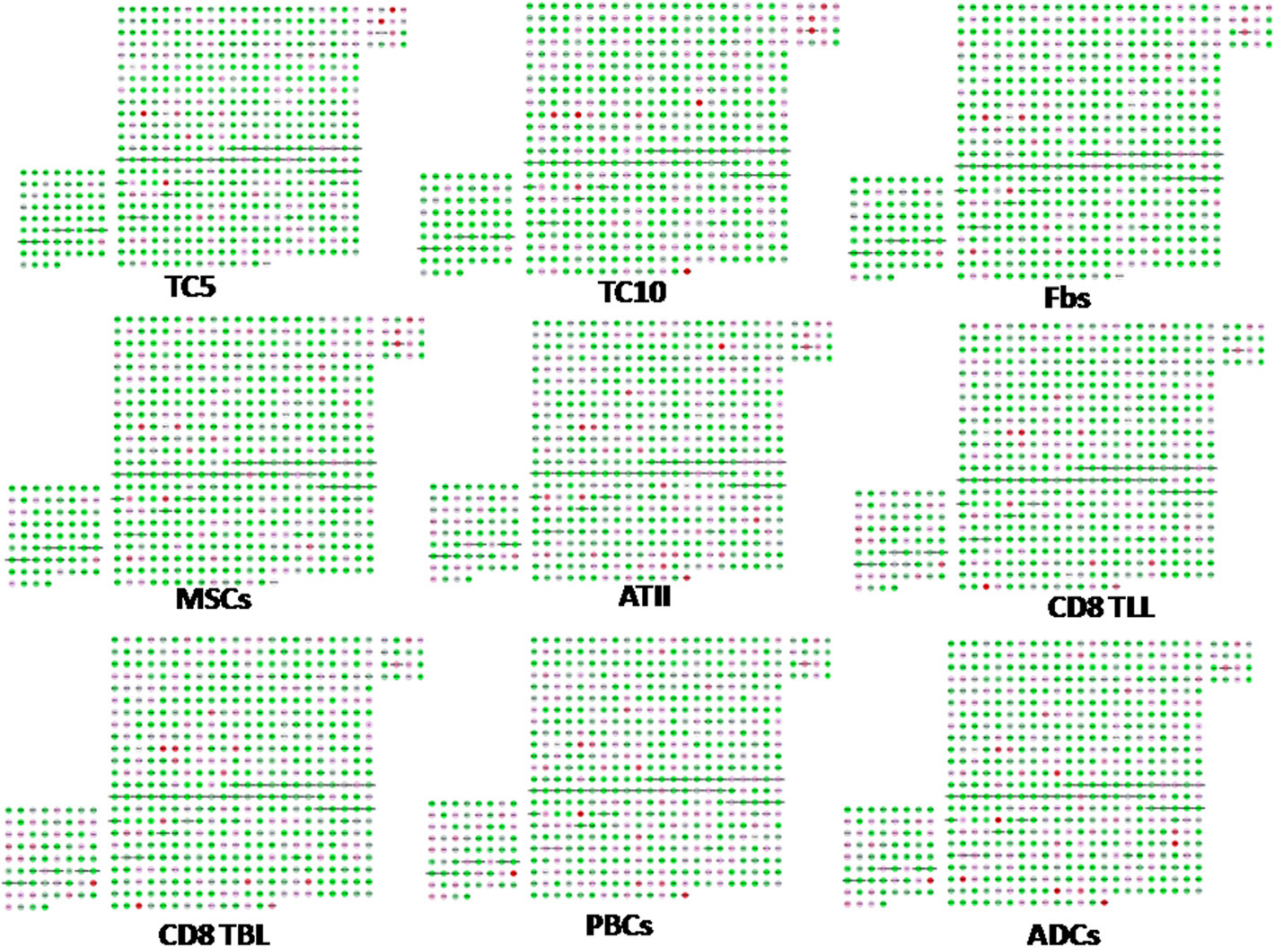
**TCs-specific genes in chromosome 17 were selected as a specific group.** Each node stands for each given gene. The upper right rectangular group represents up-regulated TCs-specific genes while the bottom left one represents down-regulated TCs-specific genes. Red nodes show genes up-regulated while green nodes show genes down-regulated.

**Figure 4:**
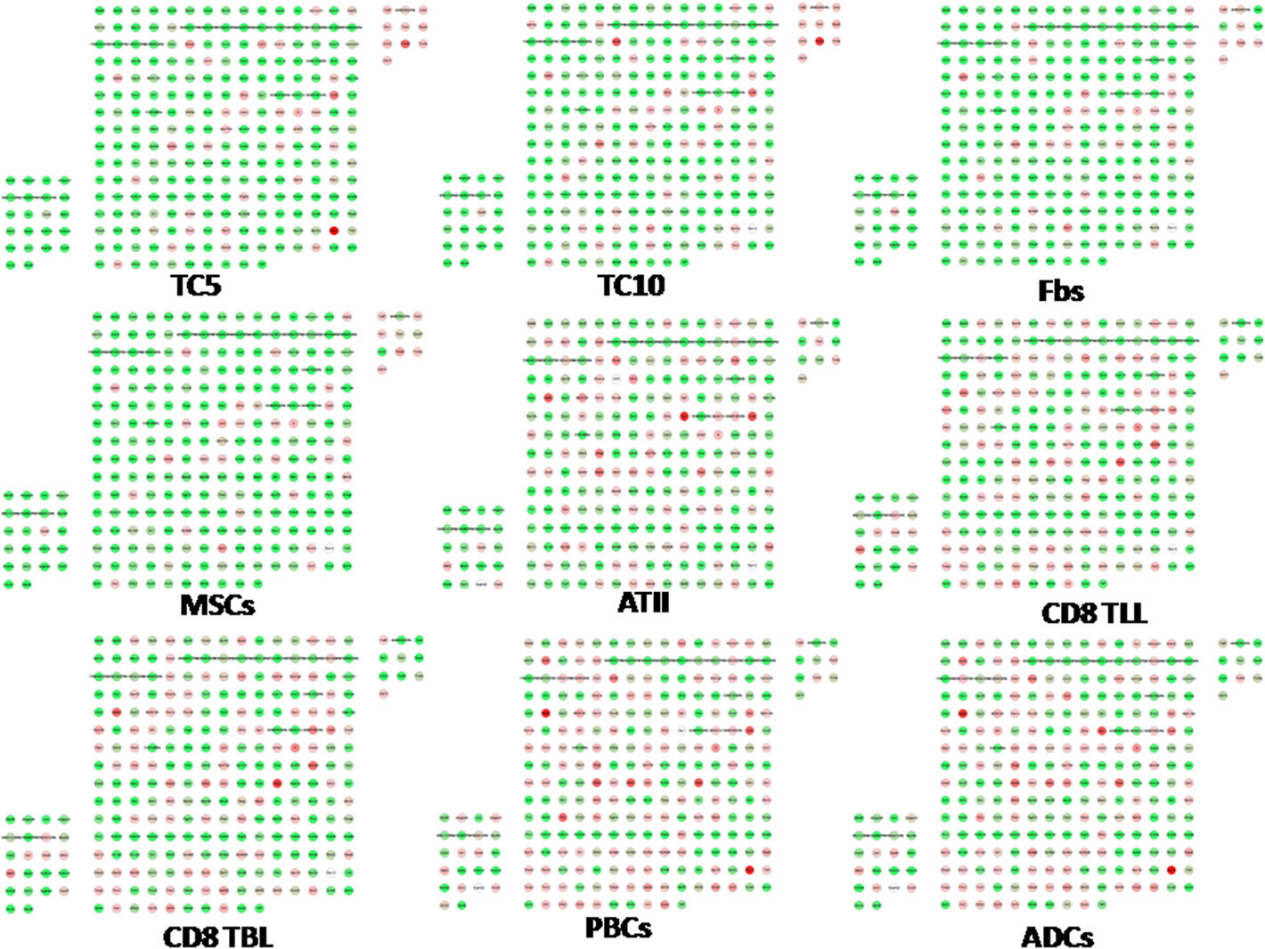
**TCs-specific genes in chromosome 18 were selected as a specific group.** Each node stands for each given gene. The upper right rectangular group represents up-regulated TCs-specific genes while the bottom left one represents down-regulated TCs-specific genes. Red nodes show genes up-regulated while green nodes show genes down-regulated.

**Figure 5:**
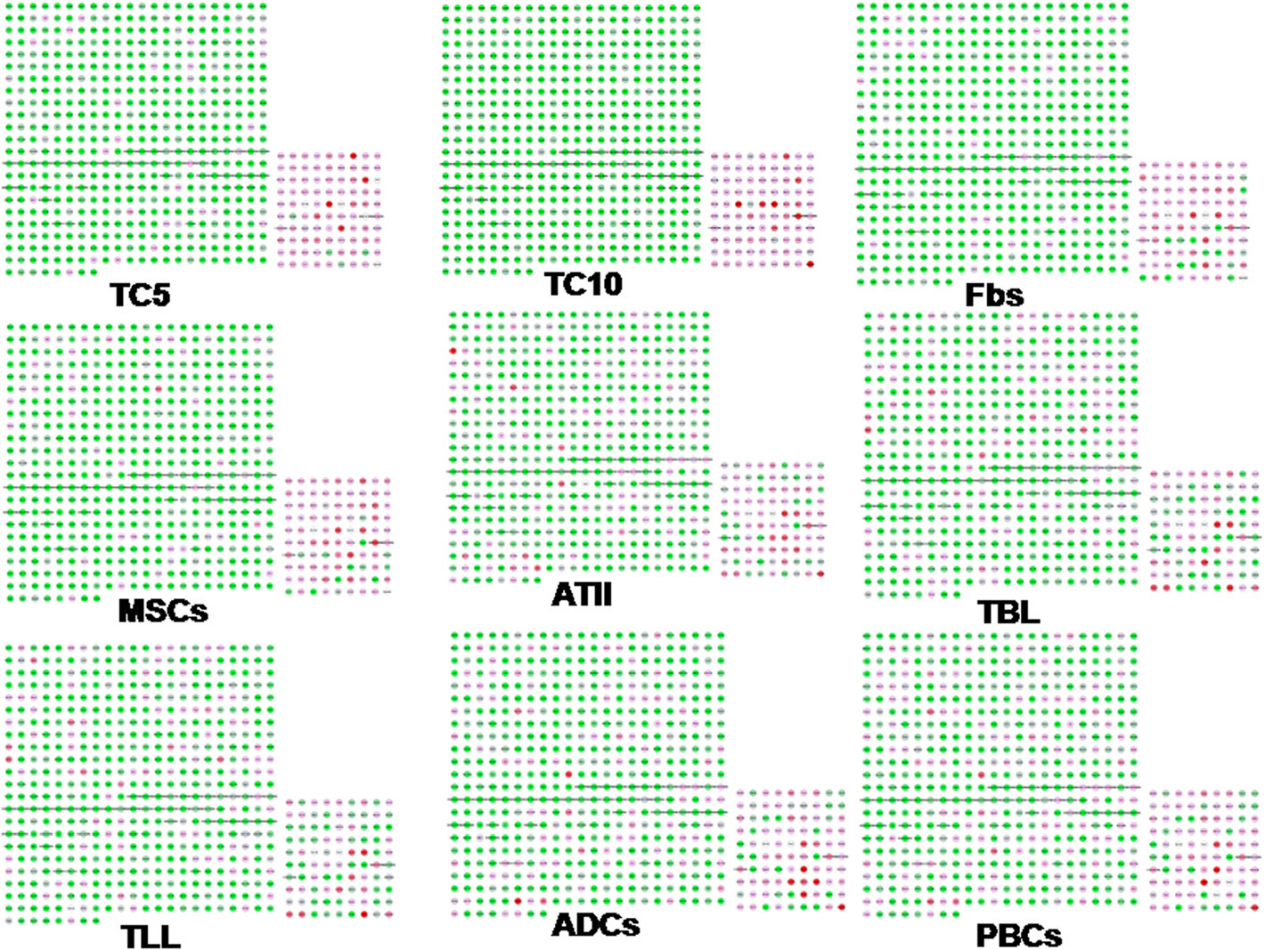
**Top 20% up-regulated genes in TC10 was extracted and compared with the other cells via the normalize gene expression data in chromosome 17.** Each node stands for each given gene. The bottom right rectangular group represents top 20% up-regulated TC10 genes.Red nodes show genes up-regulated while green nodes show genes down-regulated.

**Figure 6:**
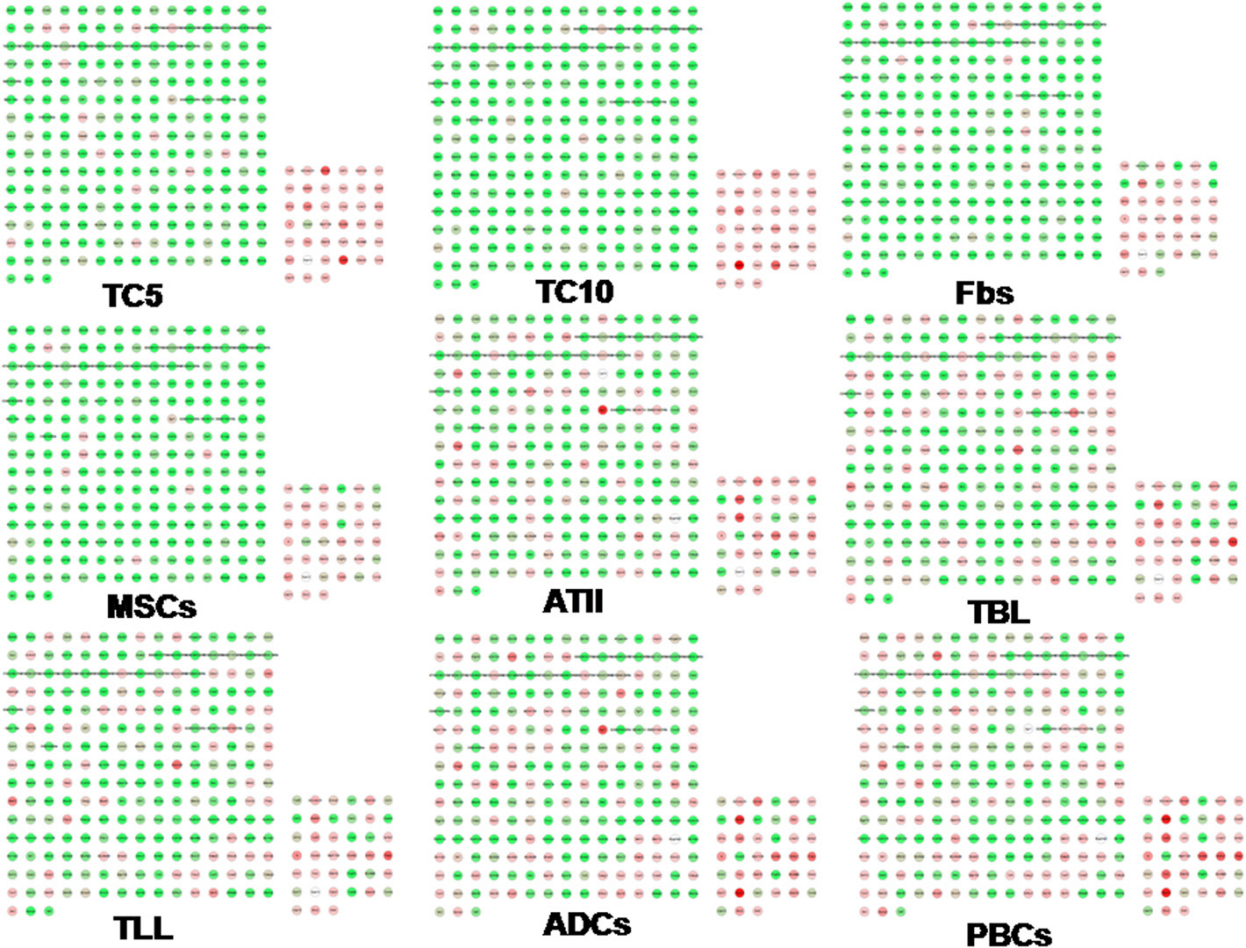
**Top 20% up-regulated genes in TC10 was extracted and compared with the other cells via the normalize gene expression data in chromosome 18.** Each node stands for each given gene. The bottom right rectangular group represents top 20% up-regulated TC10 genes.Red nodes show genes up-regulated, while green nodes show genes down-regulated.

Hierarchical clustering of genes in chromosomes 17 and 18 was performed by TIGR Multi-experiment Viewer (MeV v4.9), respectively (Figure 1A and B). The physical and functional interaction of specific genes was further evaluated by String Network analysis (www.string-db.org) in chromosomes 17 and 18 (Figure 2). Twenty six genes presented close associations with each other. TCs-specific genes which were up- or down-regulated in TC5 and TC10 were selected as gene clusters. Figures 3 and 4 demonstrated differential changes of TCs-specific genes in TCs and other cells. Top 20% up- or down-regulated genes in TC5 or TC10 were extracted and compared with other cells via the normalize gene expression data in either chromosomes 17 (Figure 5) or 18 (Figure 6). The result showed that high or low expressed genes in TCs had few similarities with Fbs, MSCs, ATII, ABCs, PACs, T-BL, or T-LL, respectively.

## Discussion

Chromosome 17 represents between 2.5 and 3 percent of human genome while chromosome 18 approximately 2.7% [30,31]. It has been reported that chromosomes 17 and 18 contained many disease-associated genes [32-37]. The present study initially demonstrated TCs-specific genes, of which 16 and 10 up-regulated and 68 and 22 down-regulated, in chromosomes 17 and 18, respectively. Of them, data from the present study indicated Mapk14, Trem2, MCFD2, or Dpysl3 as the representative of up-regulated genes and E4F1 and PDCD2 as the representative of down-regulated genes, though the further study is needed.

Mapk14 is the encoding gene of p38 to regulate different cellular functions, promote the production of pro-inflammatory cytokines, and be involved in the response to stress and metabolic pathways. p38 plays important roles in the maintenance of homoeostasis and related pathologies [38-40]. Trem2 is the member of Trem family which is highly conserved in evolution in different specials [41,42]. Trem2 negatively regulates cytokine synthesis and plays an inhibitory regulator in inflammatory response [43,44]. Telocytes were found to connect with immune cells and regulate the immune response [19]. Over-expressed Mapk14 gene in TCs may promote the production and secretion of cytokines or other signal materials to induce inflammation, while up-regulated Trem2 gene tends to inhibit inflammatory cytokine synthesis to counteract the activation of p38. It seems that TCs play a complicated and dual role in immune surveillance and immune regulation. During the physical process, TCs can be activated to maintain homeostasis to induce proliferation, differentiation and tissue regeneration. On the other hand, TCs initiate the tissue inflammation to induce pathogenesis under some challenges.

MCFD2 encodes proteins involved in the transport of FV and FVIII from the endoplasmic reticulum to the Golgi apparatus [45]. It is suggested that TCs play an important role as an autocrine/paracrine factor in maintaining stem cell potential and self-renewal [46,47]. TCs were recently discovered to join the stem cells in the regeneration and repair from myocardial infarction [48]. We speculate that MCFD2 up-regulated in TCs may promote positive interaction and intercommunication between TCs and stem cells, and contribute to tissue injury and repair by maintaining tissue homoeostasis. CRMP4 protein encoded by Dpysl3 is strongly expressed in the developing nervous system, and play a critical role in neuronal outgrowth and polarity, axon guidance and axonal protection, and regeneration [49,50]. The features of neural axon have great similarity with Tps of TCs on morphology and biological function. It is possible that up-regulated Dpysl3 is indispensable for TCs self-proliferation and cell-cell network forming.

E4F1 controls mammalian embryonic and somatic cell proliferation and survival, and is a key posttranslational regulator of p53, which modulates its effector functions involved in cell growth arrest or apoptosis [51,52]. The low expression of E4F1 in TCs has a positive effect on cell proliferation and differentiation and maintains the viability and activity of TCs in tissues. Programmed cell death 2(PDCD2) is a highly conversed protein and expressed in embryonic and adult tissues widely. The transfection of a construct expressing PDCD2 could induce apoptosis in human cell lines [53]. Equally, TCs down-regulate the PDCD2 to balance apoptosis and proliferation to maintain the homeostasis.

## Conclusion

The study globally analyzed the variation of genes in chromosome 17 and 18 among pulmonary TCs, MSCs, Fbs, ATII, ABCs, PACs, T-BL, and T-LL for the first time. Approximately 15% and 12% genes in chromosomes 17 and 18 were identified as TCs-specific genes. The specific up-regulated genes, i.e. Mapk14, Trem2, MCFD2 and Dpysl3, and specific down-regulated genes, i.e. E4F1 and PDCD2, in chromosome 17 and 18 made us have a deeper insight into biological features and functions of TCs. It has been found that TCs present morphological contact with immune cells to form a cellular interaction network to participate in immune regulation [19]. Our study revealing variation of immune-associated genes in chromosome 17 and 18 gave an additional support to the essential role of TCs in immune surveillance and immune homeostasis which may protect from immune disorder in acute and chronic pulmonary diseases. TCs also played a vital role in tissue proliferation, differentiation and regeneration to maintain the tissue homeostasis.

## Reviewers’ comments

### Reviewer 1: Dragos Cretoiu

This topic is original and the study provides new cues within the field of the telocytes (TCs), a distinct type of stromal cells. There are some minor issues which, if addressed, could improve the manuscript.

1) Introduction – There are more papers already published regarding the presence of telocytes in different organs, which might be worth mentioning. Some characteristics of the immunophenotype and ultrastructure should also be described.

**Authors’ response:** We have now described the presence of TCs in different organs and the characteristics of the immunophenotype and ultrastructure in introduction paragraph.

The aim of the study is not clearly stated and therefore a strong justification for the purpose of this study must be included. Also, some discussion about the chromosome 2 and 3 gene expression profile in TCs should be mentioned.

**Authors’ response:** We revised and highlighted the aim of our study to investigate features and patterns of TCs-specific gene profiles and explore potential function of TCs by focusing in chromosomes 17 and 18. It was not only the first time but also a new method to mine the TCs-specific gene profiles in chromosome 17 and 18. We now also have made a discussion about the study for the chromosome 2 and 3 gene expression profiles in TCs, which had not been published before we submitted this article.

2) Results – It would be useful for the readers to describe name of gene (or the encoded protein) at least for the most significant ones.

**Authors’ response:** We completely agree with this suggestion and have listed these significant genes in Tables 1, 2, 3, 4, 5, 6, 7 and 8.

3) Conclusion – I would suggest that this paragraph should be re-written pointing out the specific novelty of this study and the major findings leading to some functional hypotheses. Anyway, phrases like “Data from the present study demonstrated TC-specific genes in chromosomes 17 and 18, although the mechanism of TCs-specific genes in biological process of TCs needs to be furthermore explored” must be removed since is hampering the clarity and the significance of the study. TCs diversity according to organ location is quite intriguing, even considering the proposed roles of this cell subset in immune surveillance/tissue homeostasis. The authors should comment more on this.

**Authors’ response:** We thank the reviewer’s advice and have re-written the conclusion paragraph to highlight the specific novelty of our study and TCs-specific genes identification with its potential function in chromosomes 17 and 18. Also, negative phrases like “Data from the present study demonstrated TC-specific genes in chromosomes 17 and 18, although the mechanism of TCs-specific genes in biological process of TCs needs to be furthermore explored” have been removed.

Besides, TCs was isolated from murine lung tissue in this study. We have described the proposed roles of TCs in immune surveillance/tissue homeostasis which may protect from immune disorder in acute and chronic pulmonary diseases. We think it’s really meaning to differentiate the proposed roles of TCs subsets from different organ location in further study, as reviewer suggested.

4) An English language revision is mandatory. Many sentences should be rephrased. For example “The novel feature of telocytes under TEM has extremely long TPs with podomers and podoms, to contact with neighboring or distant effectors like axinal of neuron”.

Quality of written English: Needs some language corrections before being published.

**Authors’ response:** We have revised the article by an English native specialist. I hope you are satisfied with the revised version of the manuscript and hope it suitable for the publication.

### Reviewer 2: Qing Kay Li, Department of Pathology, Johns Hopkins Medical Institutions

Although telocytes (TCs) have been found in a variety of organs, TCs-specific biomarkers still need to be evaluated. This manuscript studied the gene expression profiles of murine pulmonary TCs and compared their profiles with a variety of cell types, including mesenchymal stem cells, fibroblasts, alveolar type II cells, airway basal cells, proximal airway cells, CD8+ T cells from bronchial lymph nodes, and CD8+ T cells from the lung. In the study, genes of chromosome 17 and 18 were extracted and analyzed using bioinformatics tools, in order to identify TCs-specific genes and their functional networks. They found that 16 and 10 of TCs-specific genes were up-regulated, and 68 and 22 were down-regulated in the chromosome 17 and 18, respectively. Particularly, Mapk14, Trem2, MCFD2 and Dpysl3 were up-regulated, whereas the E4F1 and PDCD2 were down-regulated in TCs. Their data demonstrated that the differential expression of subset of genes in chromosomes 17 and 18 were unique features of pulmonary TCs, and they might be used as biomarkers to distinguish TCs from the other type of cells. Their findings were also suggestive of that TCs might play critical roles in immune surveillance and other intracellular functions.

Quality of written English: Acceptable.

**Authors’ response:** We appreciated Dr. Qing Kay Li’s encouraging comment on this article.

## Additional files

Additional file 1:
**Data profiles for all genes in chromosome 17.**
Additional file 2:
**Data profiles for all genes in chromosome 18.**
Additional file 3:
**Details of up-regulated and down-regulated gene expression variations in chromosome 17.**
Additional file 4:
**Details of up-regulated and down-regulated gene expression variations in chromosome 18.**

## Abbreviation

ABCs: Airway basal cells
ATII: Alveolar type II cells
DPYSL3: Dihydropyrimidinase-like 3
E4F1: E4F transcription factor 1
Fbs: Fibroblasts
GEO: Gene Expression Omnibus
MAPK14: Mitogen-activated protein kinase 14
MCFD2: Multiple coagulation factor deficiency 2
MSCs: Mesenchymal stem cells
NCBI: National Center for Biotechnology Information
PACs: Proximal airway cells
PDCD2: Programmed cell death 2
T-BL: CD8+ T cells from bronchial lymph nodes
TCs: Telocytes
TC5: TCs on days 5
TC10: TCs on days 10
TEM: Transmission electron microscopy
T-LL: CD8+ T cells from lungs
Tps: Telopodes
TREM2: Triggering receptor expressed on myeloid cells 2
